# A novel robot for CT-guided bone needle insertion with a rotational drilling and force-feedback speed control mechanism: preliminary evaluation in swine

**DOI:** 10.1007/s11604-026-02030-5

**Published:** 2026-06-13

**Authors:** Yusuke Matsui, Takao Hiraki, Yuta Kimura, Takanori Sasaki, Ryutaro Matsuura, Koji Tomita, Noriyuki Umakoshi, Soichiro Okamoto, Kazuaki Munetomo, Fumiyo Higaki, Jun Sakurai, Toshihiro Iguchi, Atsushi Nakazawa, Kiyoshi Matsumiya, Takayuki Matsuno, Tetsushi Kamegawa

**Affiliations:** 1 Department of Radiology, Faculty of Medicine, Dentistry and Pharmaceutical Sciences, Okayama University, Okayama, Japan; 2 Department of Radiology, Graduate School of Medicine, Dentistry and Pharmaceutical Sciences, Okayama University, 2-5-1 Shikata-Cho, Kita-Ku, Okayama, 700-8558 Japan; 3 Collaborative Research Center for OMIC, Graduate School of Medicine, Dentistry, and Pharmaceutical Sciences, Okayama University, Okayama, Japan; 4 Department of Radiological Technology, Faculty of Health Sciences, Okayama University, Okayama, Japan; 5 Department of Radiology, Medical Development Field, Okayama University, Okayama, Japan; 6 Center for Innovative Clinical Medicine, Okayama University Hospital, Okayama, Japan; 7 Faculty of Interdisciplinary Science and Engineering in Health Systems, Okayama University, Okayama, Japan; 8 Department of Medical Technology, Faculty of Life Science, Okayama University of Science, Okayama, Japan; 9 Faculty of Environmental, Life, Natural Science and Technology, Okayama University, Okayama, Japan

**Keywords:** Robot, Needle, Bone, CT-guided, Intervention

## Abstract

**Purpose:**

To evaluate the feasibility and accuracy of computed tomography (CT)-guided needle insertion into swine bones by using a novel robotic system capable of rotational drilling.

**Materials and methods:**

This was an animal experiment using three swine. A remote-controlled robot equipped with a rotational drilling and force-feedback insertion speed control mechanism was developed for bone needle insertion. Using the robot, CT-guided insertion of a 10-gauge bone access needle was attempted in the lumbar vertebrae, ilia, and femora six times each. Needle insertion accuracy was evaluated using the angle error, which is defined as the difference between the predetermined and post-insertion needle angles on axial and sagittal CT images. The time required for needle insertion was measured. The angle error and time required for needle insertion were compared among the bones using the Kruskal–Wallis test. Adverse events were also evaluated.

**Results:**

Robotic bone needle insertion was successful in all attempts. The median axial and sagittal angle errors were 0.21 and 0.21° for the lumbar vertebrae, 0.32 and 0.13° for the ilia, and 0.65 and 0.25° for the femora, respectively. Axial angle errors were significantly different among the bone types (*p* = 0.038). The time required for needle insertion was 23.6, 21.3, and 59.7 s for the lumbar vertebrae, ilia, and femora, respectively. Time was significantly different among bone types (*p* = 0.017). No adverse events were observed.

**Conclusion:**

CT-guided bone needle insertion using a robot equipped with a rotational drilling and force-feedback insertion speed control mechanism was feasible and accurate in swine.

**Supplementary Information:**

The online version contains supplementary material available at https://doi.org/10.1007/s11604-026-02030-5.

## Introduction

Computed tomography (CT)-guided needle insertion is widely performed in various interventional procedures, including biopsy and tumor ablation. However, it has several inherent issues, including radiation exposure to both operators and patients, as well as challenges in complex needle insertions (e.g., those involving oblique needle trajectories) [1, 2]. Robotic and navigation systems have garnered significant attention as promising technologies to overcome the limitations of conventional CT-guided needle insertion [3–6]. Robots may improve accuracy, reduce radiation exposure to patients and physicians, and decrease learning curves in image-guided needle insertion [4]. Robots used for needle insertion can be categorized into two types: one aligns a needle guide to the planned trajectory to assist subsequent manual needle insertion (i.e., a needle navigation robot) [7–12], whereas the other not only adjusts the needle posture but also advances the needle (i.e., a needle insertion robot) [13–16]. Clinical studies have shown the feasibility and accuracy of robotic needle insertion in various soft tissue organs, including the lungs, liver, kidneys, and muscles [13–16].

With regard to the application of robotic technologies to image-guided bone intervention, the feasibility of robot-navigated bone biopsies has been demonstrated in previous studies [7, 17, 18]. In these studies, the robot was used only to align the needle guide, and needle insertion was performed manually by physicians through the needle guide. However, manual needle insertion into bone following robotic needle navigation is physically demanding and requires considerable time, especially in osteosclerotic lesions. In this context, a robotic system capable of needle insertion into the bone is considered more advantageous than a navigation robot. Compared to soft tissue organs, bones might be more suitable for robotic needle insertion, as they are neither subject to respiratory motion nor deformable, possibly reducing targeting errors. However, bone needle insertion faces additional technical challenges for the robot, such as the need to rotate the needle along its shaft to drill the bone [19, 20]. Onogi et al. developed a robot that rotationally oscillated a needle for bone drilling and successfully inserted needles into bone phantoms made of polyurethane [20]. However, robotic needle insertion into in vivo bones remains to be evaluated. In vivo, bone stiffness may vary depending on the bone type, necessitating different optimal needle insertion speeds. Thus, this study aimed to evaluate the feasibility and accuracy of CT-guided needle insertion into swine bones using a novel robot equipped with a rotational drilling and force-feedback insertion speed control mechanism.

## Materials and methods

This animal experiment was approved by the Institutional Animal Care and Use Committee (approval number: OKU-2022593).

### Robotic system

**Fig. 1:**
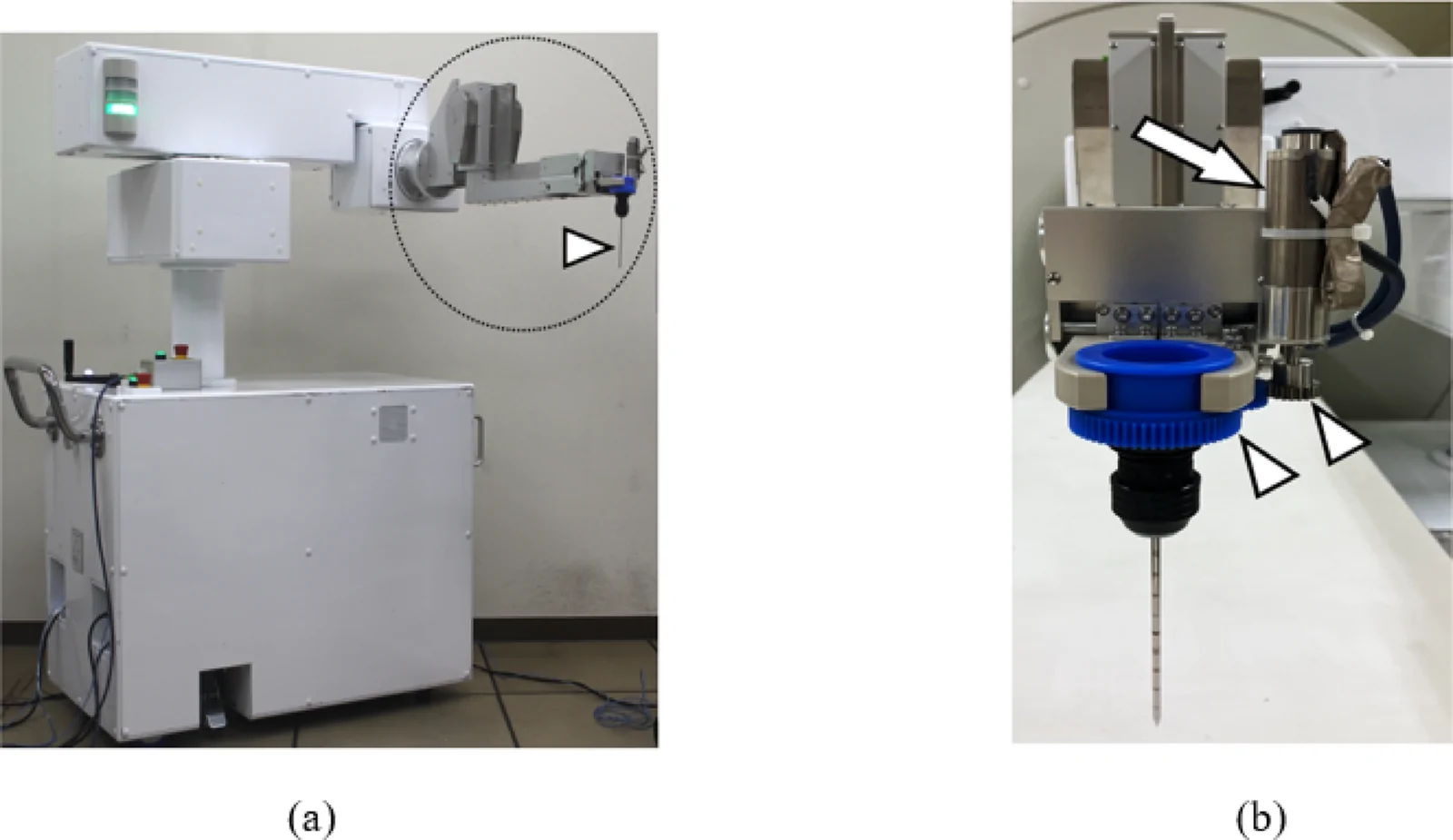
Photographs of robot used for bone needle insertion. **a** Overview of the robot. The newly developed robotic arm for bone needle insertion is indicated by a dotted circle. A commercially available bone access needle (arrowhead) is attached to the end of the arm. **b** Magnified view of the end effector. The needle may be rotated along its shaft using a motor (arrow) and gears (arrowheads) to drill into the bone

A robot that enables needle insertion into soft tissues under CT guidance (Zerobot; Medicalnet Okayama, Okayama, Japan) has been developed [21]. The robot consists of a base and manipulator parts to which an arm is attached. The robot was evaluated experimentally [22–24] and validated for various soft tissue organs in humans [15, 16]. In this study, the robot was modified to enable bone needle insertion; that is, a new arm specifically designed for bone needle insertion was developed and integrated into the manipulator (Fig. 1). A bone access needle from a commercially available bone biopsy system (Arrow^®^ OnControl^®^ Powered Bone Access System; Teleflex, Morrisville, NC, USA) [25, 26] was attached to the end effector of the arm, which was made of plastic to avoid metal artifact on CT images. Force sensors were mounted on the arm to monitor the resistance forces transmitted through the needle.

The tasks of the robot were to locate and orient the needle for targeting and then insert it. To fulfill those tasks, the robot had six degrees of freedom (DOF): two rotational DOF for needle orientation, three linear DOF for needle location, and one rotational DOF for needle rotation. Needle advancement was achieved by synchronizing the three linear DOFs. Each needle movement was performed by a physician via an operation interface, which consisted of a touchscreen console and a controller. The physician held and manipulated the controller by hand, and could immediately control the motion of each axis by pressing the buttons on the controller. The touchscreen console was used to monitor real-time information, including the resistance force during needle insertion, as well as to modify insertion parameters.

### Mechanism of robotic bone needle insertion

**Fig. 2:**
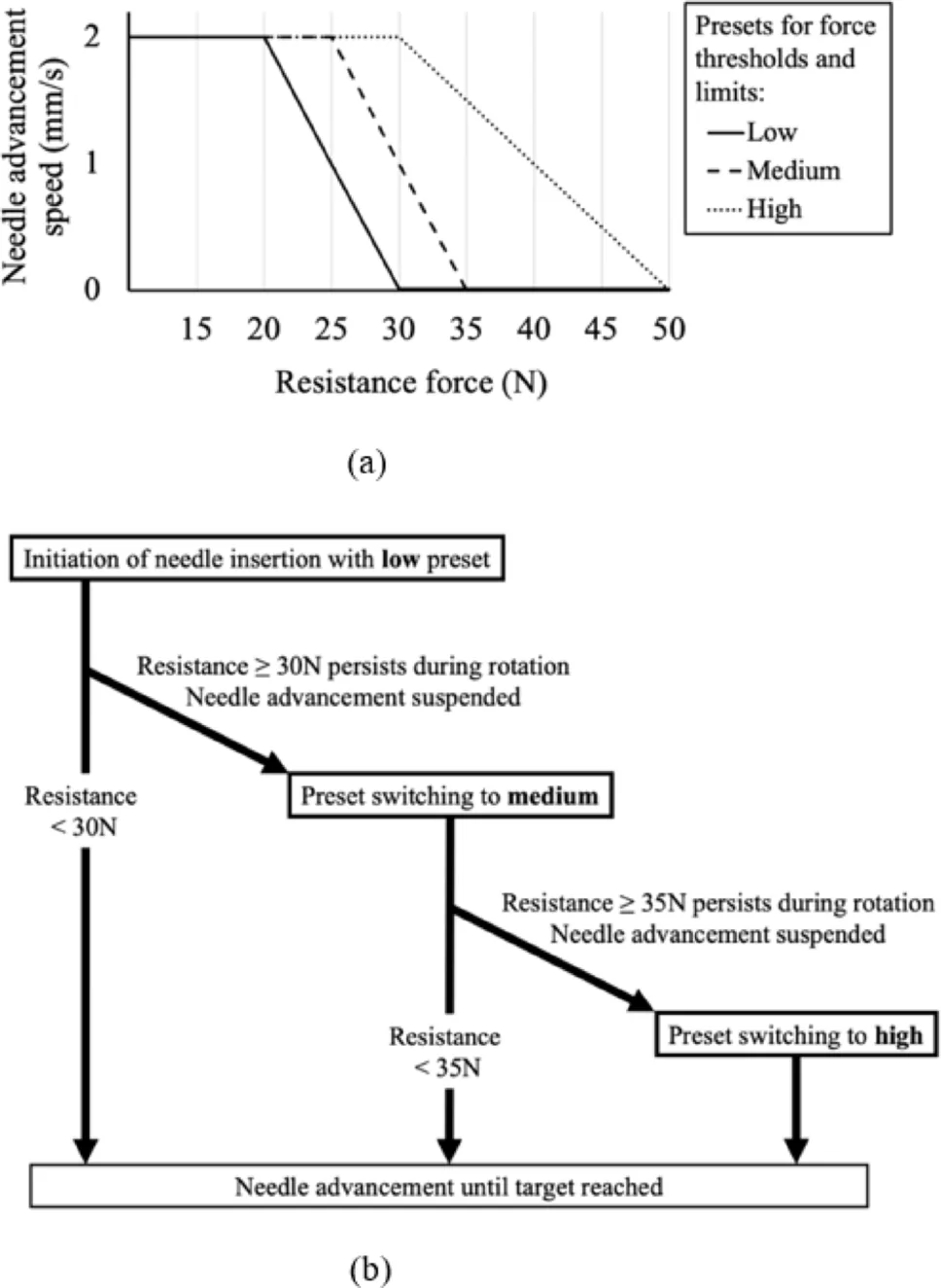
Force-feedback insertion speed control mechanism. **a** Graph showing the relationship between the resistance force to the arm and needle advancement speed. The speed was initially set to 2 mm/s and decreased linearly when the resistance force exceeded the threshold. Needle advancement was suspended when the resistance reached its limit. There were three preset thresholds and limits: low (threshold/limit = 20/30 N), medium (threshold/limit = 25/35 N), and high (threshold/limit = 30/50 N). Needle rotation was continued at 6 revolutions per second throughout the insertion. **b** Flowchart showing the switching between presets during bone needle insertion. Bone needle insertion was initiated with a low preset regardless of the bone type. If the resistance reached the limit, needle advancement was suspended while needle rotation continued. If continuous rotation caused the resistance to decrease below the limit, needle advancement automatically resumed. Otherwise, the physician manually switched the preset medium to resume needle advancement. If needle advancement was suspended, even with medium, the preset was switched to high. Needle advancement was continued until the needle tip reached the target

Needle insertion into the bones was achieved using a combination of needle rotation and advancement. The rotation speed was set to 6 revolutions per second (rps), which was similar to that of a clinically used powered driver (Arrow^®^ OnControl^®^ Powered Bone Access System) and remained constant throughout the needle insertion. By contrast, the needle advancement speed was controlled based on the resistance force measured by the sensors during insertion (i.e., force-feedback insertion speed control mechanism) as shown in Fig. 2a. Briefly, the speed was initially 2 mm/s and decreased linearly when the resistance exceeded the threshold. Needle advancement was suspended when resistance reached its limit. There were three presets (low, medium, and high) for thresholds and limits. A video demonstrating robotic needle insertion into a bone phantom is available in Supplementary Material 1.

### Animal experiment

Three male swine (weight: 35.5 to 37.5 kg) were used. All animals were healthy and had no prior experimental procedures. After the administration of intramuscular atropine sulfate, the swine were sedated using intramuscular ketamine, medetomidine, and midazolam. General anesthesia was maintained with intratracheal isoflurane while monitoring vital signs. The swine were placed in the prone position and fixed with belts on a CT table (Eminence STARGATE; Shimadzu, Kyoto, Japan).

The robotic insertion of a 10-gauge, 10-cm bone access needle was attempted six times each in the lumbar vertebrae, ilia, and femora (18 attempts in total); these three types of bones were used to evaluate the performance of the robot on bones with different properties, such as stiffness and shape. As this was an exploratory feasibility study, no control group was included. The sample size was determined pragmatically, and no formal sample size calculation was performed. An experienced interventional radiologist remotely operated the robot using an interface while continuously monitoring axial CT images of 4-mm thickness on the CT console screen.

After performing CT of the target bone, a skin incision was made at the entry point of the needle. First, the needle was oriented to the target bone under CT guidance, and then the needle was advanced using the robot until its tip reached the bone surface. Next, the needle insertion trajectory in the bone was determined on the CT console screen, with the trajectory angle set to 90 ± 30° relative to the bone surface. The physician then placed a cross-shaped pointer on the bone along the planned needle trajectory on the screen, which served as the target point for insertion. The distance between the bone surface and the target point was set at 10–30 mm.

**Fig. 3:**
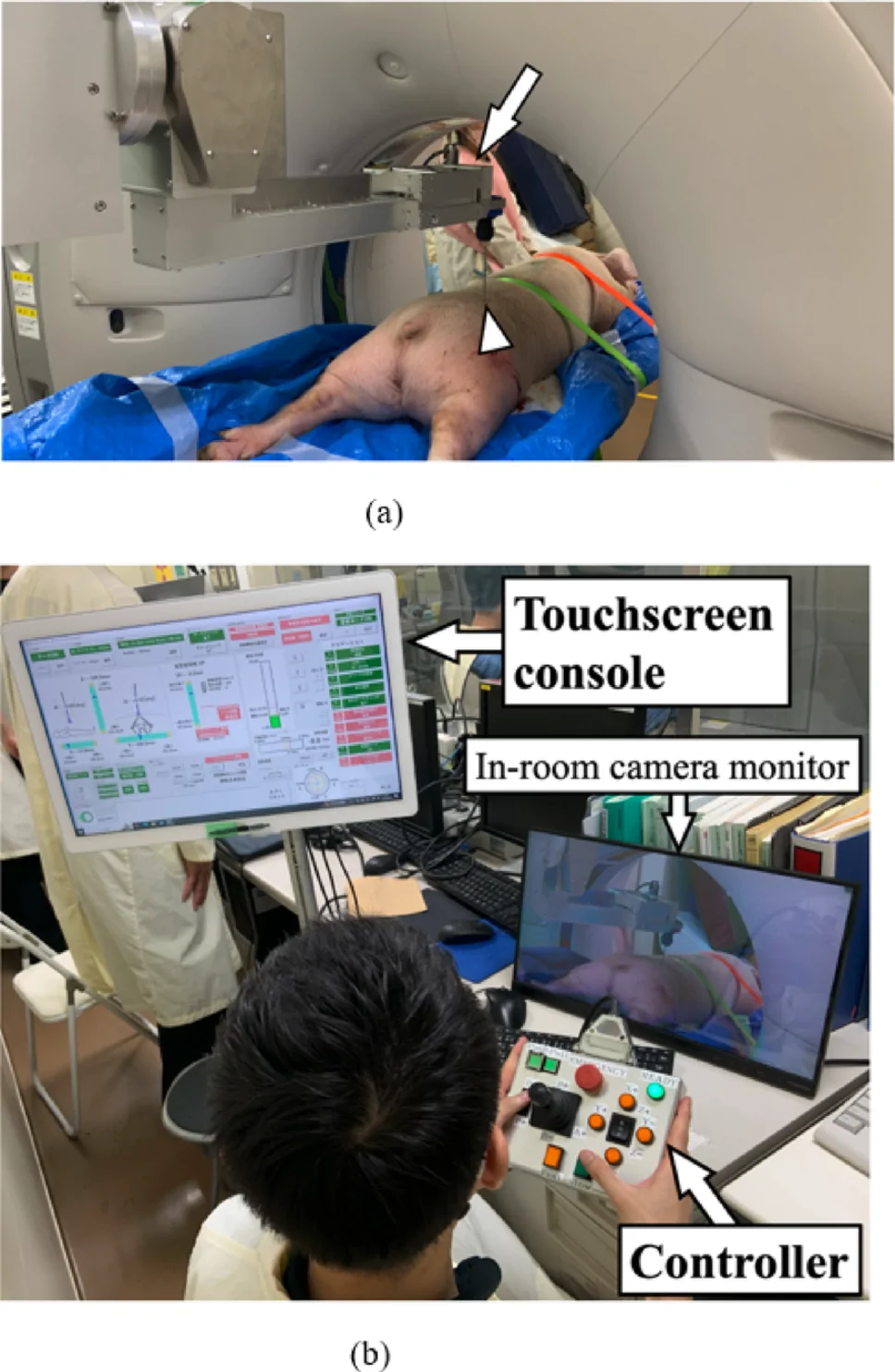
Robotic bone needle insertion in swine. **a** Photograph captured in the CT room. The swine is placed in the prone position and fixed with belts on a CT table. The robotic arm (arrow) holding the bone-access needle (arrowhead) with an end effector is positioned in the CT gantry. **b** Photograph captured outside the CT room. A physician remotely operates the robot using an interface comprising a touchscreen console and controller. The touchscreen console displays various real-time information for needle insertion, including the needle angle and position, as well as the resistance force. During needle insertion, the physician monitored both the operative field displayed using an in-room camera and CT images of 4-mm thickness (not shown in this figure) continuously displayed on the CT console screen

The needle was inserted into the bone under continuous real-time monitoring of single-slice CT images using the dynamic mode, with the following imaging parameters: tube voltage, 120 kV; tube current, 50 mA; and rotation time, 0.5 s. The details of robotic needle insertion with the rotational drilling and force-feedback insertion speed control mechanism are shown in Fig. 2b. Once the needle tip reached the target point, the insertion was stopped. Subsequently, CT images of the target bone were obtained to assess the needle insertion accuracy and adverse events. The time required for the bone needle insertion was measured. Photographs taken during the animal experiment are shown in Fig. 3.

### Evaluation

Bone needle insertion was considered successful when completed without needle slippage on the bone surface, major needle deviation (i.e., advancement of the needle in a direction substantially deviating from the planned trajectory, as subjectively judged by the operator), or robot malfunction. Needle insertion accuracy was evaluated using the angle error, which was defined as the difference between the predetermined and post-insertion needle angles on axial and sagittal CT images [9, 20, 24]. The length of the needle tract in the bone was measured using CT images after needle insertion. All measurements were performed by another radiologist, using a DICOM viewer (OsiriX MD version 11.0.4; OsiriX Foundation, Geneva, Switzerland). Further, the distance errors between the needle tip and the target point were estimated based on the angle errors and needle tract lengths; the estimation method is presented in Supplementary Material 2. Adverse events were evaluated by monitoring the vital signs and reviewing the CT images after needle insertion.

### Statistical analysis

A total of 18 needle insertion attempts were analyzed (n = 6 per bone type), and no data were excluded. Numerical values are summarized using the median, interquartile range (IQR), and range. The angle error and time required for bone needle insertion were compared among the three types of bones using the Kruskal–Wallis test, followed by post hoc pairwise comparisons with Bonferroni adjustment. A *p*-value of < 0.05 was considered statistically significant. The statistical analyses were performed using SPSS version 29 (IBM Corp., Armonk, NY, USA), and box plots were generated using R version 4.6.0 (The R Foundation for Statistical Computing, Vienna, Austria) and EZR version 1.70 (Jichi Medical University, Tochigi, Japan) [27].

## Results

The robotic bone needle insertion was successful in all 18 attempts. Only the low-preset force-feedback insertion speed control mechanism was used for all insertions into the lumbar vertebrae and ilia. Regarding insertion into the femora, only the low preset was used for two attempts; the preset was switched to medium for another two attempts and further to high for the remaining two attempts.

**Fig. 4:**
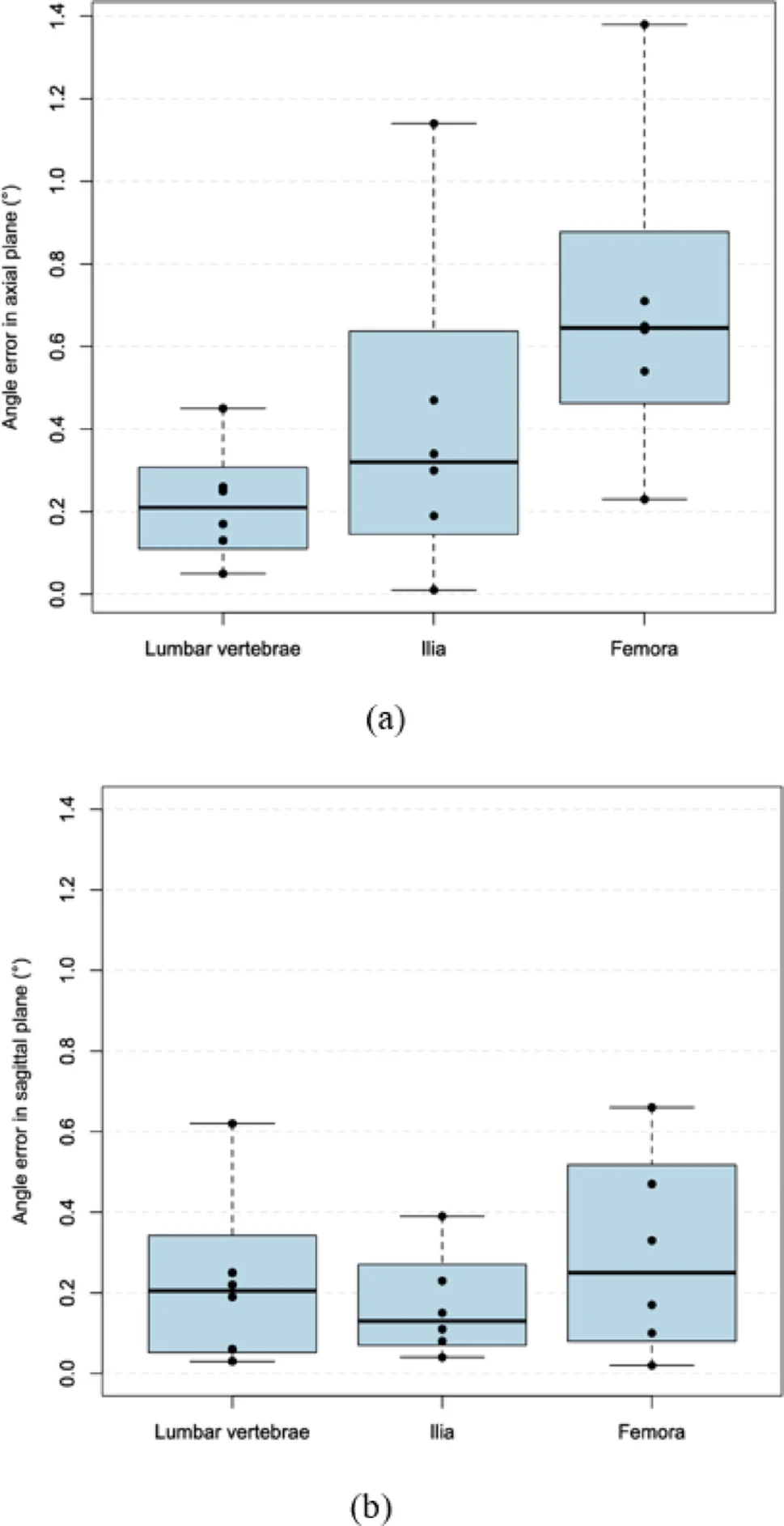
Angle errors. **a** The median axial angle errors were 0.21° (interquartile range [IQR], 0.11–0.31°; range, 0.05–0.45°) for the lumbar vertebrae, 0.32° (IQR, 0.15–0.64°; range, 0.01–1.14°) for the ilia, and 0.65° (IQR, 0.46–0.88°; range, 0.23–1.38°) for the femora. **b** The median sagittal angle errors were 0.21° (IQR, 0.05–0.34°; range, 0.03–0.62°) for the lumbar vertebrae, 0.13° (IQR, 0.07–0.27°; range, 0.04–0.39°) for the ilia, and 0.25° (IQR, 0.08–0.52°; range, 0.02–0.66°) for the femora

The median needle tract length in the bones was 10.6 mm (IQR, 10.3–13.1 mm; range, 10.1–14.1 mm) for the lumbar vertebrae, 17.7 mm (IQR, 13.6–20.8 mm; range, 13.3–22.5 mm) for the ilia, and 16.7 mm (IQR, 15.5–20.5 mm; range, 14.5–20.8 mm) for the femora. The angle errors for each bone are shown in Fig. 4. The median axial/sagittal angle errors were 0.21/0.21° for the lumbar vertebrae, 0.32/0.13° for the ilia, and 0.65/0.25° for the femora (*p* = 0.038 for the axial error and *p* = 0.751 for the sagittal error). Post hoc pairwise comparisons revealed a significant difference in the axial angle error between the lumbar vertebrae and femora (*p* = 0.033), whereas no significant differences were observed between the other pairs. The median estimated distance error was 0.05 mm (IQR, 0.05–0.09 mm; range, 0.04–0.12 mm) for the lumbar vertebrae, 0.13 mm (IQR, 0.07–0.21 mm; range, 0.05–0.27 mm) for the ilia, and 0.24 mm (IQR, 0.13–0.30 mm; range, 0.11–0.43 mm) for the femora.

**Fig. 5:**
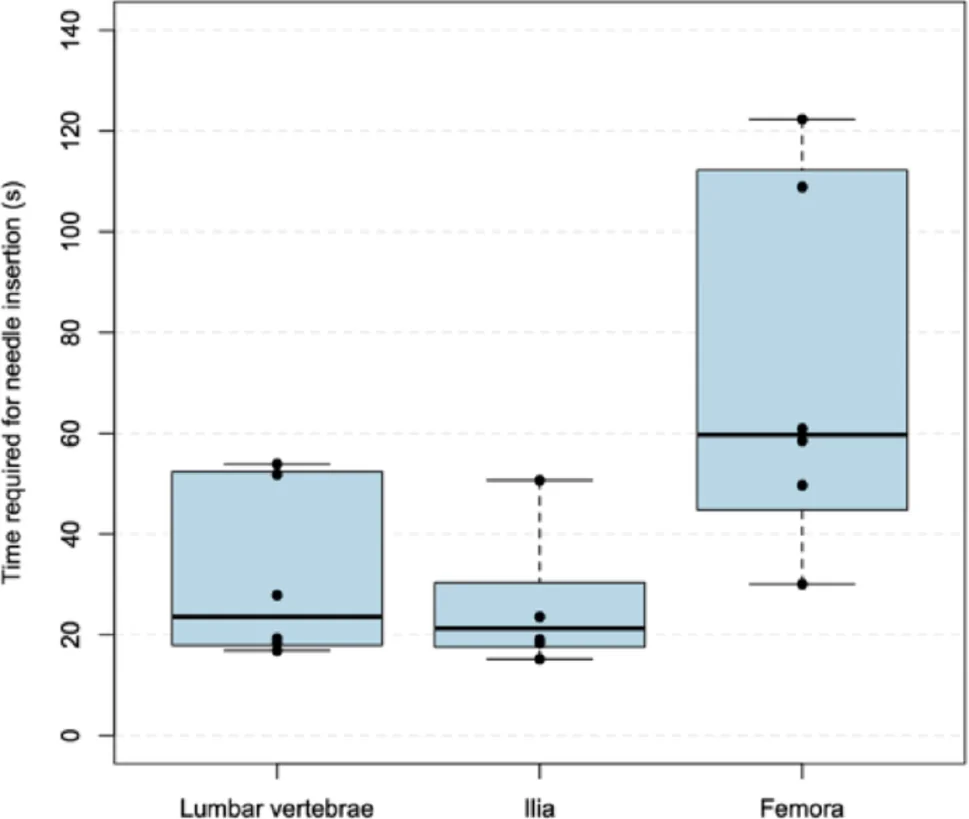
Time required for needle insertion. The median time for needle insertion was 23.6 s (interquartile range [IQR], 17.9–52.3 s; range, 16.8–53.9 s) for the lumbar vertebrae, 21.3 s (IQR, 17.5–30.4 s; range, 15.2–50.7 s) for the ilia, and 59.7 s (IQR, 44.8–112.2 s; range, 30.0–122.3 s) for the femora

The time required for needle insertion is shown in Fig. 5. The median time was 23.6 s, 21.3 s, and 59.7 s for the lumbar vertebrae, ilia, and femora, respectively (*p* = 0.017). Post hoc pairwise comparisons revealed a significant difference in the time required for needle insertion between the ilia and femora (*p* = 0.021), whereas no significant differences were observed between the other pairs.

**Fig. 6:**
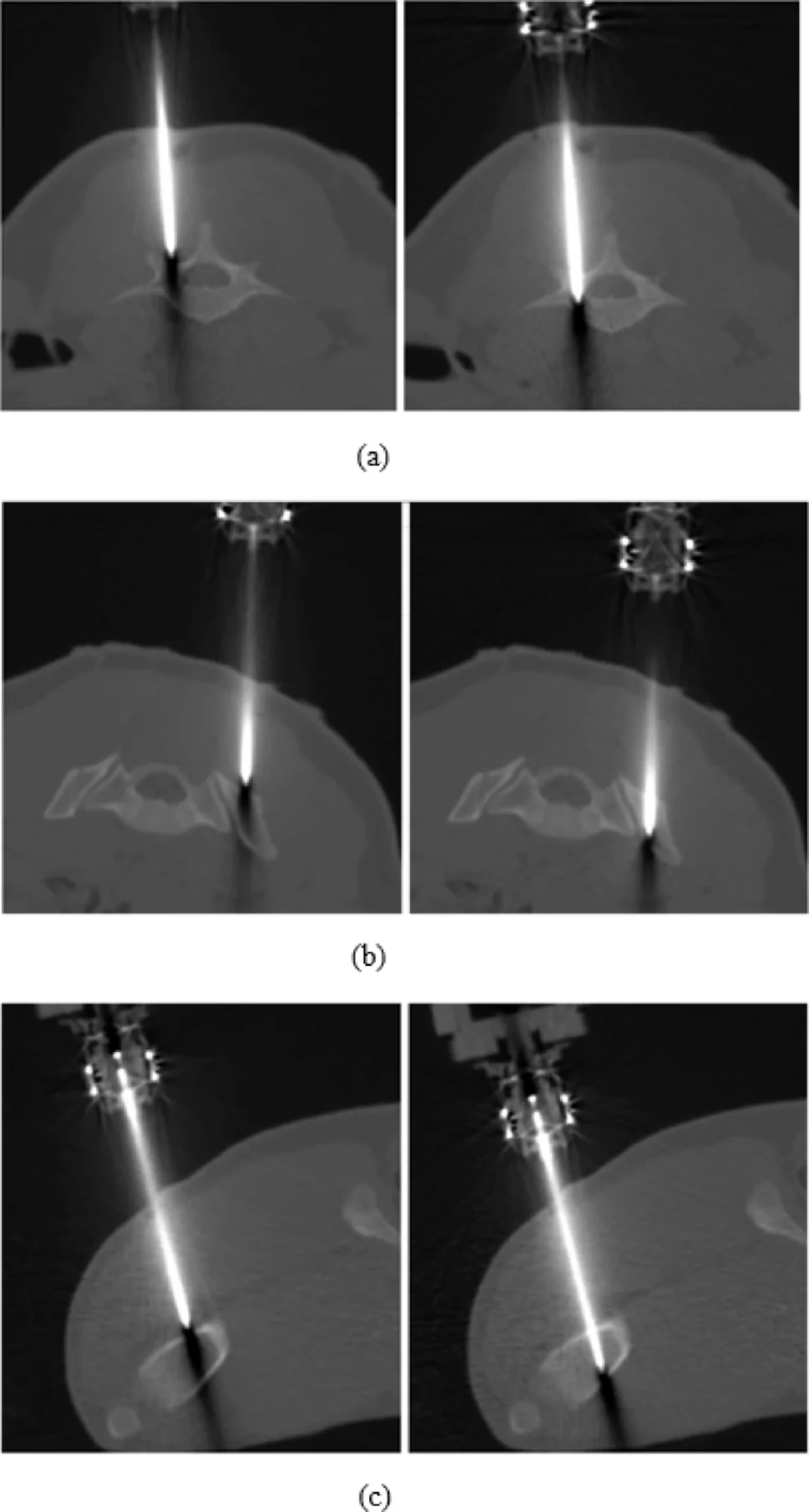
Axial CT images of robotic needle insertion into swine bones. The left and right panels show the images before and after needle insertion, respectively. **a** Lumbar vertebra (angle error = 0.05°). **b** Ilium (angle error = 0.30°). **c** Femur (angle error = 0.65°)

No adverse events were observed. Figure 6 shows the CT images before and after needle insertion into the lumbar vertebra, ilium, and femur. A video demonstrating robotic needle insertion into the ilium is available in Supplementary Material 3.

## Discussion

This study showed that CT-guided needle insertion into swine lumbar vertebrae, ilia, and femora using the newly developed robot was feasible and accurate, with median angle errors of far less than 1°. In this study, the angle error was used instead of the distance error (i.e., the distance between the target and the needle tip) to evaluate needle insertion accuracy. This method was chosen because the needle tract length in swine bones was relatively short; in such cases, the distance error might understate the actual deviation, leading to an underestimation of the error. In bone needle insertion, once the needle tip penetrates the bone cortex, the needle becomes fixed to the bone and the needle angle cannot be altered anymore. Consequently, the angle error remains constant regardless of the tract length. Therefore, the angle error serves as a more robust metric, and distance error can be estimated from the angle error and the needle tract length. Based on the angle errors and tract lengths in this study, the maximum distance error was estimated to be less than 0.5 mm. When targeting the center of a 1-cm bone lesion, the lesion can theoretically be reached if the accuracy is within 5 mm. Therefore, robotic bone needle insertion with the present system is expected to be highly accurate in clinical settings, suggesting its potential utility in various procedures, including biopsy, tumor ablation, vertebroplasty, balloon kyphoplasty, and screw fixation.

Previous studies have demonstrated the feasibility of robot-navigated bone biopsy [7, 17, 18]. Witkowska et al. reported a technical success rate of 100% for CT-guided, robot-navigated bone biopsies in 40 patients [18]. The robot held the needle steady, possibly contributing to safe and accurate insertion. However, manual bone drilling using a K-wire was required for bone biopsies [18]. Bonnet et al. recently demonstrated the safety and feasibility of robot-navigated needle insertion into bone in a preclinical study using swine [28]. They used a robotic system modified for skeletal procedures, with a thicker and more robust body and a stronger jaw spring to ensure secure and stable holding of the needle. However, needle insertion into bone was still performed manually using a mallet [28].

The robot developed in the present study is distinct from previously reported needle navigation robots in that it enables robotic needle insertion while the operator remains seated, simply by pressing buttons on the controller. This novel feature may help reduce the physical burden on physicians and minimize insertion errors associated with manual manipulation. Needle insertion into bone requires a rotational drilling mechanism, unlike insertion into soft tissues, which can be achieved by simply advancing the needle forward. The key parameters for bone needle insertion are the speeds of needle rotation and advancement. Although faster rotation results in more powerful drilling, it may pose a higher risk of tissue damage by generating frictional heat. For safety, the needle rotation speed in this study was set to be similar to that of a powered driver used in a clinical setting. Faster needle advancement may reduce the insertion time. However, if the needle is advanced too quickly before the tip is anchored to the bone surface, it may slip and deviate from its planned trajectory. In addition, fast needle advancement can generate an excessive resistance force on the robot arm, leading to the robot stopping because the robot arm cannot move as instructed (i.e., a servo error). In fact, during the early stage of developing our robot, advancing the needle at a constant speed occasionally resulted in insertion failure due to needle slippage or servo errors. Therefore, a force-feedback insertion speed control mechanism was developed to enable feasible and accurate needle insertion. The parameters used in this mechanism were based on unpublished internal data obtained from previous preliminary experiments.

In our robot, the end effector holding the needle had a slight rotational play, which may have caused a needle angle error. Furthermore, the slight displacement of the target bone during pushing with the needle may have contributed to the angle error. The larger axial angle error in the femur may be attributable to less fixation of the lower limb compared with the lumbar vertebra or ilium. The longer time and frequent manual switching of presets required for insertion into the femur may be explained by the thicker and harder cortex, which leads to a higher force and thereby decreased advancement speed. To address this, an automated preset-switching system is currently under development.

Robotic needle insertion with the present system has several potential advantages and concerns compared with manual needle insertion. The median time required for needle insertion was less than one minute for each type of bone, suggesting that robotic bone needle insertion might be time-efficient. However, the robotic procedure requires additional time for setting up the robot, which may prolong the total procedure time. A notable benefit of robotic needle insertion is that physicians can avoid radiation exposure by operating the robot remotely from outside the CT room. Other theoretical advantages include a shorter learning curve and thereby improved procedural reproducibility with less dependence on physician skill. However, as this study did not include a direct comparison between manual and robotic needle insertion, further comparative studies are warranted to thoroughly evaluate these advantages. Heat generation during needle rotation and the associated risk of thermal damage to bone and surrounding soft tissues may represent a potential concern in robotic bone needle insertion. Compared with manual drilling, in which physicians may intermittently adjust the drilling force and rotation, robotic drilling may be more continuous, potentially increasing the risk of frictional heat accumulation. Therefore, incorporating a temperature-monitoring mechanism may be considered a potential future technical improvement.

This preliminary study had several limitations. This was an animal experiment using a small number of swine. Because the number of needle insertion was small, the statistical power is limited, and the results of the statistical analyses should be regarded as exploratory in nature. Because no physical intrabony marker was used as a target, the measured angle error may not fully represent true targeting accuracy. Nevertheless, the narrow range of the angle errors (within approximately 1°) suggests high reproducibility of accurate needle insertion with the robot. Swine bones differ from human bones in terms of bone composition, density, and mechanical strength [29]; therefore, the results of this study cannot be directly generalized to humans. Needle insertion was performed under limited conditions. All procedures were performed by a single physician using a single type of commercially available bone needle. Only short (10–30 mm) needle tracts were allowed because the swine were small in size. The needle trajectory angles were within 90° ± 30° relative to the bones. Therefore, further investigation is needed to evaluate the feasibility of robotic needle insertion with longer or more complex trajectories. In addition, only three types of healthy bone were targeted; needle insertion into bones with osteosclerotic lesions might be more challenging. Unlike the animal experiment under general anesthesia, clinical bone interventions are often performed under local anesthesia, during which patient movement may occur. Therefore, safety measures for patient movement warrant further investigation before clinical application. Finally, this study focused on the feasibility and accuracy of robotic needle insertion, and bone specimen acquisition was not attempted despite the use of a bone access needle from a biopsy system. Future studies should evaluate the feasibility of bone biopsy using the robot, including the assessment of specimen quantity and quality.

## Conclusion

A novel robot capable of bone needle insertion for CT-guided intervention was developed. Needle insertion using the robot equipped with the rotational drilling and force-feedback insertion speed control mechanism was feasible and accurate for swine bones.

## Supplementary Information

Below is the link to the electronic supplementary material.

Supplementary Material 1. Video demonstrating robotic needle insertion into a bone phantom

Supplementary Material 2. Method for estimating distance error in robotic bone needle insertion

Supplementary Material 3. Video showing robotic bone needle insertion into the ilium of a swine
