# Supplementary material for: A novel robot for CT-guided bone needle insertion with a rotational drilling and force-feedback speed control mechanism: preliminary evaluation in swine
Source: Jpn J Radiol. 2026 Jun 13;44(10):1836–44. doi: 10.1007/s11604-026-02030-5 (PMC13624013; doi:10.1007/s11604-026-02030-5)
Supplement: Supplementary file 1 [file 11604_2026_2030_MOESM2_ESM.pdf]

Supplementary Material 2. Method for estimating distance error in robotic bone needle insertion

The distance error between the needle tip and the target point was estimated based on the predetermined and post-insertion needle angles and the needle tract length. The estimation method followed that used in a previous study [24]. Because the angle error observed in the present study was small, the post-insertion needle tract length was assumed to be equal to the predetermined needle tract length. In addition, it is assumed that there is no error at the needle entry point.

A unit vector (e_x, e_y, e_z) representing an arbitrary needle direction in three-dimensional space is given using the needle angle on the axial plane θ_x and that on the sagittal plane θ_z, as follows.

$$e_y = \sqrt{\frac{1}{(1 + \tan^2\theta_x + \tan^2\theta_z)}}$$

$$e_x = -e_y \tan\theta_x$$

$$e_z = e_y \tan\theta_z$$

Let the predetermined needle direction be (e_x^*, e_y^*, e_z^*), and the post-insertion needle direction be (e_x^a, e_y^a, e_z^a). The angle ϕ between the two unit vectors was determined using the following equation.

$$\phi = \cos^{-1}\left(e_x^* e_x^a + e_y^* e_y^a + e_z^* e_y^a\right)$$

Then, the distance error d was estimated from ϕ and the needle tract length L using the following equation.

$$d = 2L\sin\left(\frac{\phi}{2}\right)$$
